# Detection of equine atypical myopathy-associated hypoglycin A in plant material: Optimisation and validation of a novel LC-MS based method without derivatisation

**DOI:** 10.1371/journal.pone.0199521

**Published:** 2018-07-03

**Authors:** Sonia González Medina, Carolyne Hyde, Imogen Lovera, Richard J. Piercy

**Affiliations:** 1 Comparative Neuromuscular Diseases Laboratory, The Royal Veterinary College, London, United Kingdom; 2 Bio-Analysis Centre, London, United Kingdom; College of Agricultural Sciences, UNITED STATES

## Abstract

Hypoglycin A (HGA) toxicity, following ingestion of material from certain plants, is linked to an acquired multiple acyl-CoA dehydrogenase deficiency known as atypical myopathy, a commonly fatal form of equine rhabdomyolysis seen worldwide. Whilst some plants are known to contain this toxin, little is known about its function or the mechanisms that lead to varied HGA concentrations between plants. Consequently, reliable tools to detect this amino acid in plant samples are needed. Analytical methods for HGA detection have previously been validated for the food industry, however, these techniques rely on chemical derivatisation to obtain accurate results at low HGA concentrations. In this work, we describe and validate a novel method, without need for chemical derivatisation (accuracy = 84–94%; precision = 3–16%; reproducibility = 3–6%; mean linear range R^2^ = 0.999). The current limit of quantitation for HGA in plant material was halved (from 1μg/g in previous studies) to 0.5μg/g. The method was tested in *Acer pseudoplatanus* material and other tree and plant species. We confirm that *A*. *pseudoplatanus* is most likely the only source of HGA in trees found within European pastures.

## Introduction

Hypoglycin A (HGA), L-methylenecyclopropyl alanine, is a naturally-occurring but non-proteic amino acid of certain plants with unknown biological function [1]. However, the toxic effect of its active metabolite has been demonstrated and studied in several animal species, including humans and horses [2–7]. The presence of HGA is a characteristic of trees and shrubs of the *Sapindaceae* family [8, 9]. Among them, Ackee and Lychee are pertinent to human health and food industries, due to associated fatal disease outbreaks [4, 10–12]. In recent years, the link between HGA toxicity and a common, acquired, multiple acyl-CoA dehydrogenase deficiency of horses has been identified [6, 7, 13–15]. This form of rhabdomyolysis is known as atypical myopathy or pasture-associated myopathy, and its high mortality [16, 17] has revived interest in HGA and its detection.

Early techniques used for HGA detection in plant tissues relied on paper chromatography [8, 18, 19] and later, on ion-exchange chromatography [20]. Both techniques presented several problems in the separation of HGA from similar amino acids such as leucine and isoleucine that were invariably present in samples, leading to compromised precision and accuracy. The use of reverse phase, liquid chromatography and pre-column derivatisation with o-phthaldialdehyde (OPA) in the presence of 2-mercaptoethanol slightly improved the technique’s resolution [21, 22], however it was the use of phenylisothiocyanate (PITC) as a derivatising agent that yielded the best results allowing optimal separation of closely-related amino acids [23]. Further optimisation and validation of the proposed method by Ware (2001) determined the official use of this chromatographic method for analysis of HGA in Ackee products [24, 25]. However, this method still had a high limit of quantification (1.4μg/ml). Since HGA is potentially toxic at lower concentrations [2], several authors have described new methodologies that led to reductions to this limit, by using liquid chromatography coupled with mass spectrometry (LC-MS) technology, and derivatisation with dansyl chloride to measure HGA, as well as other downstream metabolic analogues such as methylenecyclopropyl-glycin (MCPG) [9, 26]. Derivatisation is commonly used in chromatographic methods as it can reduce selectivity issues and improve stability and the separation from other closely-related compounds. Further, it improves detection, by increasing the signal-to-noise response [27–29]. However, it also increases cost and considerably lengthens sample preparation time [27]. Here we describe a novel method using LC-MS that yields good results when analysing plant material without the use of any derivatising agent and with an improved quantification limit of 0.5μg/g.

## Methods

### Materials and standards

Hypoglycin A (purity >95%) was obtained from Toronto Chemicals (Toronto, Canada) while 3-fluorovaline was obtained from Apollo Chemicals (Stockport, UK). LCMS Optima grade solvents used for buffers were purchased from Fisher Scientific (Loughborough, UK). HPLC grade water for dissolution and methanol for extraction was purchased from VWR (Lutterworth, UK). Individual stock solutions (1mg/ml) of HGA and 3-fluorovaline were prepared by dissolving 1mg of each compound in 1ml of HPLC-grade water. The standard working solutions were obtained by diluting the stock solution at the concentration needed with water and stored at 4°C. Natural hazelnuts (*C*. *avellana*), obtained from a local market, were used as a matrix-matched plant material to perform the validation. Seeds and plant materials tested from other species were obtained from local pastures and parkland by the researchers.

### Sample preparation

Twenty-five grams of hazelnuts were homogenised to a fine powder with a 500W grinder device (Tefal-Minipro). Then HGA was added to exactly 1g of seed homogenate at the appropriate concentration (calibration curve standards 0.5–25μg; validation controls 0.8;8;24μg; blanks 0μg). Validation control samples were prepared in triplicate. 4ml of methanol (MeOH) was added to each sample and heated for 1h at 50°C. Samples were subsequently spun at 4800G for 15 min and the supernatant transferred to clean tubes and evaporated at 50°C under a nitrogen stream. The pellet was reconstituted with 100μl internal standard solution (3-fluorovaline at 5μg/ml in H_2_O) and 900ul of deionised water (DI) of LC-MS grade. The samples were then mixed by vortex for 30 seconds before transfer to glass HPLC vials.

### LC−MS/MS

HGA concentrations in spiked hazelnut homogenates were determined on an LCMS-8040 triple quadrupole instrument with a Nexera LC front end (Shimadzu UK, Milton Keynes) using positive-mode ESI. Samples were injected at 1μL volumes onto a Phenomenex Kinetex 2.6μ HILIC 100A (50 x 2.1mm) column at 25°C temperature. Mobile phases consisted of 2mM ammonium formate (pH3) in DI (buffer A) and 0.1% Optima grade formic acid in Optima grade acetonitrile (buffer B). A gradient was delivered at 0.4ml/min starting from 95% buffer B for 0. 5 min, followed by a decrease to 50% over 2 mins, held at 50% for 1 min, then reduced to 30% buffer B over 0.1min and held at 30% for 1 min before returning to 95% buffer B over 0.1 min for re-equilibration for 2.3mins. The following optimised instrument parameters were applied for the detection of the analytes: nebulising gas at 3L/min; drying gas at 15L/min; heat block temperature at 400°C; desolvation line temperature at 250°C; Column oven at 25°C; Autosampler temperature at 10°C. The complete system was controlled by LabSolutions software (Shimadzu), version 5.65, running on a HP Prodesk computer with Windows 7 operating system.

Quantitation was determined by multiple reaction monitoring (HGA quantitation ion m/z 142.2 → 74.05, Dwell time 100ms; Q1 Pre-bias at -15.0V; Collision Energy at -10V; Q3 Pre-bias at -29.0V. FVal quantitation ion m/z 136.2 → 70.0, Dwell time 100ms; Q1 Pre bias -14.0V; Collision Energy at -16.0V; Q3 Pre-bias at -28.0V.

### Optimisation of extraction technique

Methanol, aqueous ethanol and water extraction have all been suggested as solvents of choice for HGA extraction [9, 26, 30, 31]. Carlier *et al*. (2015) also proposed the use of sonication to improve and perhaps shorten the extraction time from ackee fruit. We tested 3 different solvents (methanol, ethanol and water) at 50°C for 1h on 4 different sycamore seed homogenates and compared them to the extraction obtained on duplicate samples with the same solvents at room temperature (20°C) for 24h. Results were analysed by ANOVA followed by Fisher’s LSD multiple comparison test.

### Evaluation of plant matrix

Seed homogenates were evaluated as matrix-matched material to produce the calibration standards for the methodology. Seeds from field maple (*A*. *campestre)*, Norway maple *(A*. *platanoides)*, Ash tree *(Fraxious excelsior)*, each that have seeds that are morphologically similar to those of sycamore trees (*A*. *pseudoplatanus*) were tested alongside hazelnuts (*C*. *avellana*). Two seed homogenates from 2 different trees of each species, were spiked with 10μg HGA, then extracted (as above). Recovery obtained in the 4 different seed matrices was compared by ANOVA using Dunnett’s multiple comparison test. Differences were considered statistically significantly different when p<0.05.

### Evaluation criteria for validation

Criteria used in this work followed the National Measurement System Guidelines of the United Kingdom [32] and the European Medicines Agency [33] and are summarised below. Validation was performed over 5 days during which calibration curves and validation controls were freshly prepared 3 times on 3 different days by 2 different analysts.

1. Accuracy: Calculated as the mean of the inter-day theoretical recovery at each concentration of the hazelnut homogenate validation control (VC) samples, lower validation control (LVC), middle validation control (MVC) and upper validation control (UVC). Accuracy was considered acceptable if the recovery was between 80% and 110% of the designated HGA concentration.
2. Precision: calculated as the coefficient of variation (CV) of the intra-day accuracy of fortified hazelnut homogenate VC samples. Precision was considered acceptable if the percent CVs for the 3 repeated measurements were less than 15%, except at the LVC concentration where it could be ≤20%.
3. Reproducibility: calculated as the CV of the inter-day accuracy of fortified hazelnut homogenate VC samples. Reproducibility was considered acceptable if the percent CVs did not exceed the level calculated by the Horwitz equation (Hweq) CV = 2^(1–0.5logC)^, where C is the concentration of the analyte as a decimal fraction.
4. System Linearity: determined by the evaluation of 3 HGA standard curves prepared and analysed on 3 separate days. Each standard curve consisted of 8 standards ranging from 0.1μg/ml to 25μg/ml equivalent concentration. For each validation run, the resulting HGA standard curve was expected to meet the following conditions:
  - The deviation of the calculated concentration of each standard should be within ±15% of the theoretical value (±20% at LLOQ).
  - Individual standards (≤1/3) could be dropped from the curve if they did not meet these criteria, although no quantitation should be extrapolated outside the range covered by the acceptable standards.
5. The Lower Limit of Detection (LOD): determined from 6 blanks according to the following formula: LOD = mean concentration response + (3 x Std. Dev.)
6. Limits of Quantitation: The lower limit of quantitation (LLOQ) and the upper limit of quantitation (ULOQ) were established for the method and defined as the lowest and highest validated concentration that met all acceptance criteria. Accuracy and Precision was established at the LLOQ.
7. Stability of Processed Samples:
  4°C: re-injection stability was assessed by repeat injection of a complete set of aged standard samples after storage at 10°C for 5 days. The re-injection run was considered successful if the mean concentration of the stability samples was within ±15% of the mean of freshly-prepared samples of the same theoretical concentration.
  -20°C and freeze/thaw: The stability was considered acceptable if the mean of the freeze/thaw samples (stored at -20°C) was within ±15% of the mean of freshly- prepared samples. Calibration standards were evaluated over 2 months, at day 34 and 62 after preparation. The samples went through 6 freeze-thaw cycles between day 1 and 34 and 3 freeze-thaw cycles between day 34 and day 62. Values obtained were further evaluated by ANOVA to detect any significant difference between measurements.
8. Analysis of Other Plant Samples:
  Several plant samples were selected on the basis of their common occurrence in or near UK pastures and similarity or possible confusion with sycamores, their species, or their known toxicity in horses. They included sycamore and other *Acer* trees (*A*. *pseudoplatanus*, *A*. *campestre*, *A*.*platanoides*) horse chestnut (*A*. *hippocastanum)*, ash (*F*.*excelsior*), ragwort (*J*. *sylvestris*), common mallow (*M*. *sylvestris)*, oak (*Q*. *robus*), yew (*T*. *baccata)* and beech (*F*. *sylvatica*).

## Results

**Fig 1:**
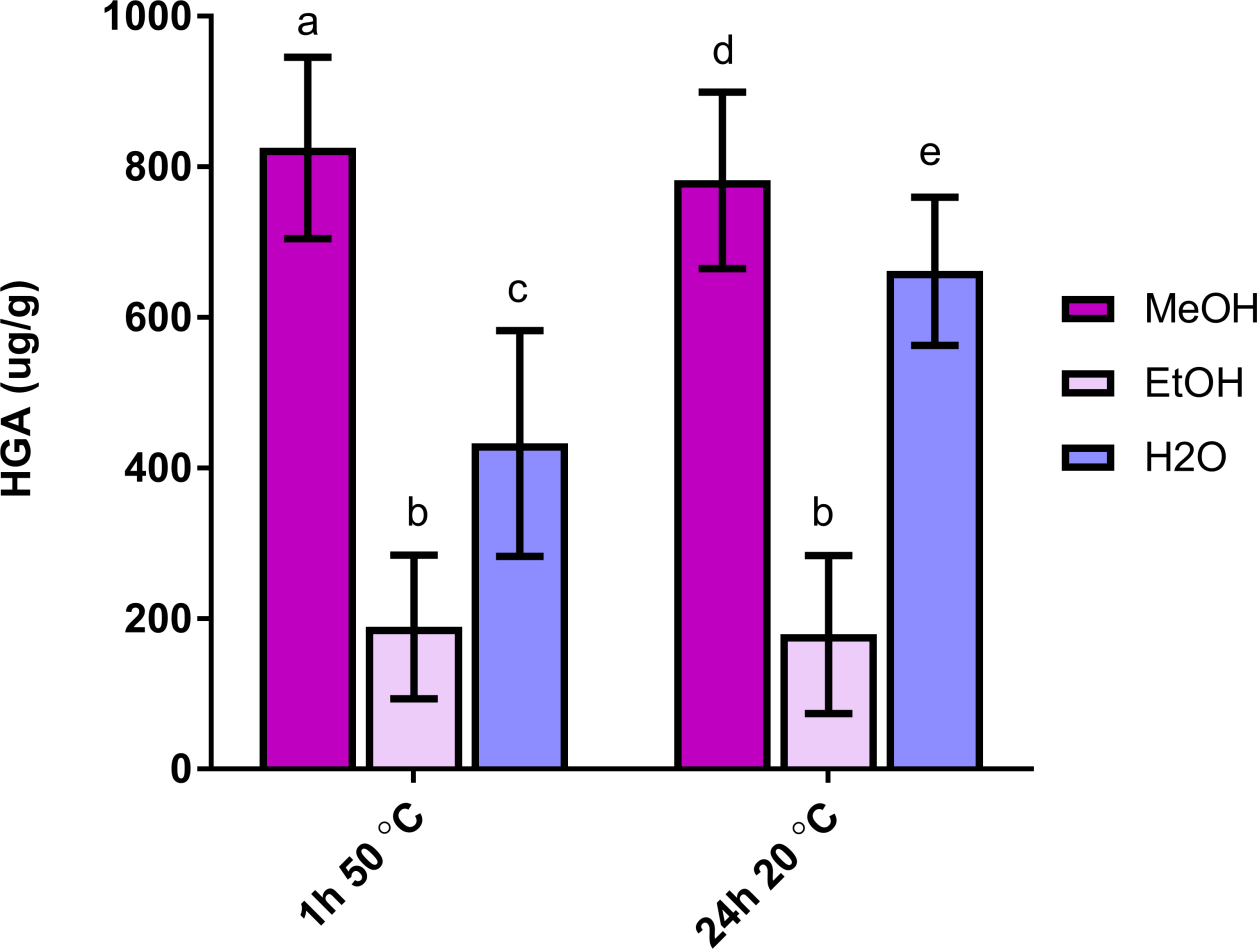
Results obtained in the extraction experiment, represented as mean ± SD of 6 seed homogenates exposed to 3 different solvents at 2 different temperatures and durations. MeOH yielded the best results at both temperatures and ethanol showed consistently low extraction performance. Aqueous extraction improved with time. Bars with different lower case letters are statistically significantly different: a-b p = 0.004; a-c p = 0.006; a-d p = 0.04; a-e p = 0.22; b-e: p = 0.01.

Each of the different solvents resulted in different degrees of extraction, with the methanol solvent performing best p<0.001 (Fig 1). There was no statistically significant difference between the 2 methanol extractions conducted under the 2 conditions (p = 0.5). In contrast, MeOH extraction was significantly better than both water and EtOH extraction at 50°C for 1 h (p = 0.008 and <0.001 respectively). MeOH extraction at 20°C for 24 hours was significantly better than the equivalent EtOH sample (p<0.001), but there was no statistically significant difference between the MeOH and water sample extracted for 24h (p = 0.10) (Fig 1). As a result, MeOH was used as extraction solvent, for 1 hour at 50°C for all subsequent experiments.

**Fig 2:**
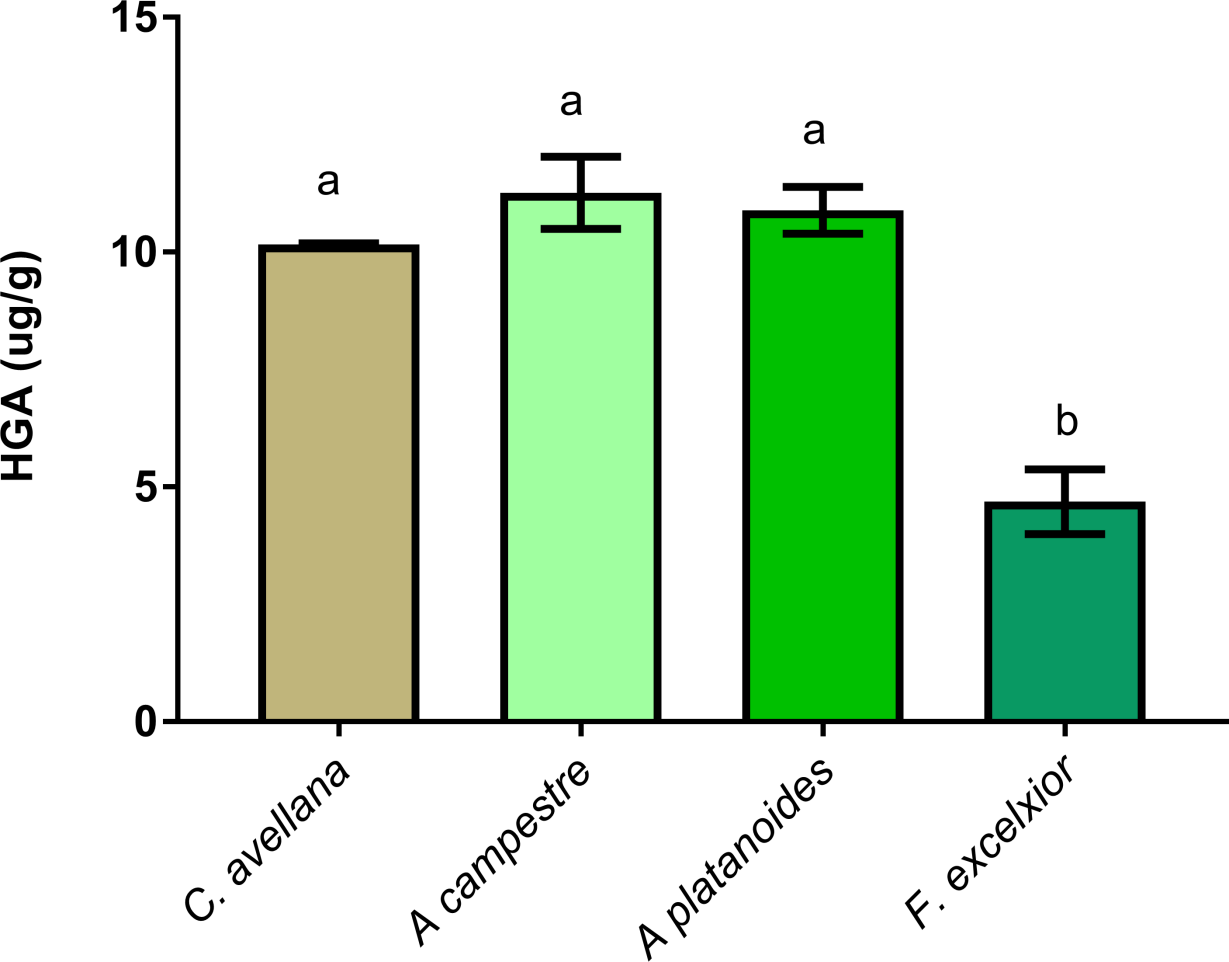
Matrix-matched experiments testing HGA-spiked extracts added to seeds from four different trees: C avellana (hazelnut), A. campestre (field maple), A. platanoides (Norway maple) and Fraxious excelxior (ash tree). Results are presented as mean ±SD of HGA measurements in two different seed matrices/species. There was no statistically significant difference in recoveries obtained from each of the first 3 tree seed homogenates, however ash tree showed poor recovery when spiked with HGA. Hazelnut was finally used for method validation as it is easy to obtain in local markets without need for field collection. Bars with different lower case letters are statistically significantly different (p = 0.001).

There was no statistically significant difference between spiked HGA recoveries from maple tree seeds and hazelnuts, but recovery was significantly lower in ash seed homogenates (46.84% ± 6.89) when compared to the other seed matrices (p<0.001) (Fig 2). As a result, hazelnut homogenate was chosen for the validation protocol.

The requirements of the validation protocol were met for the 3 concentrations evaluated (validation controls). Precision was acceptable with an intra-day coefficient of variation ranging from 3.44–11.11% for MVC and UVC and 5.65 to 15.85% for LVC. Reproducibility met the criteria of the Hortwitz equation for each concentration evaluated (Table 1). System linearity was acceptable between 0.5μg/ml and 25μg/ml (Table 2) as the deviation from the nominal value was higher than 20% for the lower concentrations (0.1μg/ml and 0.25μg/ml). The limit of detection for the method was established at 0.001μg/ml (n = 6; mean = -0.001 ± 0).

**Table 1:** Data obtained from analysis of the results for the validation controls: Upper validation control (UVC = 24μg/ml); middle validation control (MVC = 8μg/ml) and lower validation control (LVC = 0.8 μg/ml).

VALIDATION PARAMETERS		LVC	MVC	UVC
		0.8μg/ml	8μg/ml	24μg/ml
Accuracy	Inter-day Recovery (%)	94.13	92.24	84.29
Precision	Day 1 Intra-day CV (%)	7.08	6.40	3.44
	Day 2 Intra-day CV (%)	5.65	3.86	8.00
	Day 3 Intra-day CV (%)	15.85	11.11	8.56
Reproducibility	Horwitz Equation Results (%)	16.5	11.7	7.0
	Inter-day CV (%)	3.84	5.18	3.67

Notice that accuracy meets the validation criteria (recovery 82–101%). Precision was well below the 15–20% Coefficient of variation (CV) established and reproducibility of the method did not reach the Horwitz equation threshold.

**Table 2:** Linear correlation of analytes derived from freshly prepared samples during the validation process.

	μg/ml	0.1	0.25	0.5	1	2.5	5	10	25
set 1	mean	0.1275	0.27	0.49	0.99	2.44	4.94	10.14	24.96
R^2^ = 0.9999	recovery	127.5	107.2	97.2	99.15	97.44	98.83	101.36	99.86
	% from nominal	-27.5	-7.2	2.8	0.85	2.56	1.17	-1.36	0.14
set 2	mean	0.183	0.31	0.54	1.01	2.54	4.92	9.72	25.12
R^2^ = 0.9998	recovery	183.5	122.6	108.6	101.2	101.66	98.4	97.23	100.48
	% from nominal	-83.5	-22.6	-8.6	-1.2	-1.66	1.6	2.76	-0.48
set 3	mean	0.17	0.29	0.51	1.04	2.50	4.97	9.76	25.10
R^2^ = 0.9998	recovery	169	118.4	103	104.5	99.84	99.39	97.605	100.398
	% from nominal	-69	-18.4	-3	-4.5	0.16	0.61	2.395	-0.398

Notice that the concentration range between 0.5*μ*g/ml and 25*μ*g/ml met the validation requirements: recovery = 82–102% and deviation from nominal <20% for the lower limit. Quantitation range for the method was established between 0.5–25*μ*g/ml (highlighted in green). Values that are outside the acceptable range (>20% from nominal) are highlighted in yellow.

**Fig 3:**
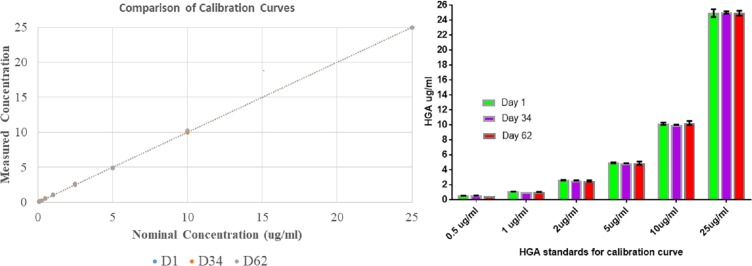
Results are presented as mean ±SD of duplicate injections. Calibration standards were evaluated at day 1 (D1), day 34 (D34) and day 62 (D62) after undergoing 9 freeze-thaw cycles. There was no statistically significant difference between any of the measurements, showing that the calibration standards are stable at -20°C for at least 2 months and that they maintained HGA concentrations after several freeze-thaw cycles.

Short-term stability of extracted samples was good at 10°C, with the percentage difference between results obtained on the first day and the subsequent measurements (day 4 and day 5) ranging from -0.07% to -8.59%. Continuing tests have shown that the calibration curve samples are remarkably robust (data not shown). Analysis of the calibration curve from day 1 on days 34 and 62 showed no significant differences (Fig 3).

Of all the plant samples analysed, only sycamore plant material was demonstrated to contain HGA (Table 3).

**Table 3:** Results are presented as median (range) for HGA concentration obtained in different plant tissues.

Species tested	HGA concentration in Seeds (median, range)	HGA concentration in Leaves (median, range)	HGA concentration in Seedlings (median, range)
Sycamore (n = 9) (*Acer pseudoplatanus)*	64.47μg/g (34.6–505.8)	43.3 μg /g (6.8–134)	1210 μg /g (264–2703)
*Norway maple* (n = 2) *Acer platanoides*	<LOD	<LOD	N/A
*Field maple* (n = 2) *Acer campestre*	<LOD	<LOD	N/A
*Horse chestnut (n = 2)* *(Aesculus hippocastanum)*	<LOD	<LOD	N/A
Ash tree (n = 2) *(Fraxious excelsior)*	<LOD	<LOD	N/A
Oak (n = 2) *(Quercus robus)*	<LOD	<LOD	N/A
Beech (n = 2) *(Fagus silvatica)*	<LOD	<LOD	N/A
*Common mallow* *(Malva sylvestris)* (n = 2)	<LOD	<LOD	N/A
Ragwort (n = 4) *(Jacobea vulgaris)*	<LOD	<LOD	N/A
Yew (n = 1) (Taxus baccata)	<LOD	<LOD	N/A

Notice that only sycamore tissues contained HGA of those analysed for this work. <LOD = below limit of detection (<1ng/g). N/A = not analysed

## Discussion

This work provides new methodology to reliably quantify HGA in plant material, lowering the current limit of quantitation of published methods [9, 24] and avoiding the use of a derivatising agent that would usually increase the sample preparation time and cost of the test. Additionally, this work demonstrates that sycamores are most likely the only source of HGA in European pastures.

A rapid and accurate test for plant-derived toxins might be helpful diagnostically, in particular, when horse or human health is at risk. By avoiding need for derivatisation, the assay can be performed in under 2.5h. Further, derivatisation can lead to uncontrollable reaction recovery and subsequent purification procedures can reduce the method’s reliability [27]. However, the authors acknowledge that chemical derivatisation is a useful technique that can increase the sensitivity of some analytical methods, particularly when lower detection limits are desired and when the chemical characteristics of the analyte are a constraint [27–29]. However, this work demonstrated that detection of HGA in plant material does not require derivatisation to produce reliable, reproducible and sensitive results; indeed, we achieved a lower limit of quantitation for HGA of 0.5μg/g in plant material which halves the previous limit of 1μg/g reported by Isenberg *et al*. 2016 [9]. Our method should enable more accurate characterisation of this toxic compound in plant material, which is of paramount importance to the food industry and horse owners, enabling, amongst other experiments, future study of the mechanisms that lead to differences in HGA concentration between plants and toxin survival following plant desiccation and herbicidal treatments.

The method we report is the first technique—validated to industry standards—that is suitable for use in measuring HGA concentration in Sycamore tree seeds; further, we show that it is reliable for use with other plant material including leaves, seedlings and nuts. The results obtained in sycamore plant material are in line with previous studies [6, 13, 26, 34], although the authors of these previous studies did not describe whether the methods used were with a fully-validated analytical method, or performed to industry standards. *Westermann et al*. *2016*, also used a related LC/MS method without derivatisation to measure HGA in Acer extracts; however a full description of the validation used in that work was not reported [34]. Current analytical methods for detection of HGA were validated for edible fruits [9, 24], however due to the impact of HGA intoxication in equine medicine, we believed that validation with sycamore seeds was necessary to guarantee the test’s performance in this plant material and provide quality data in the future study of equine disease outbreaks. In contrast to previously validated methodology [9], we did not include MCPG (recently linked to a toxic encephalopathy syndrome in humans [12, 35, 36], due to lack of commercially-produced MCPG as a standard. Still the relevance of this compound in human intoxication is not well understood as both compounds, HGA and MCPG are present in lychee fruit [9, 36, 37] and metabolites of both amino acids seem to be present in human patients [35, 36]. MCPG disrupts some fatty acid metabolic pathways [38–40] downstream to those inhibited by HGA, but it has also been suggested to act as a precursor of HGA in plant tissues [41]. The relevance of MCPG as well as hypoglycin B (HGB) in horse outbreaks is yet to be investigated. We believe that expansion of the current method to include MCPG, HGB and other hypoglycin isomers might be technically feasible when pure analytical standards are commercially available.

In addition to method validation, this work provides information regarding sources of HGA in European pastures. A limited sampling of other tree species commonly present in/or surrounding grazing areas as well as two plant species were investigated. Other than Sycamore trees, none of the analysed material contained hypoglycin A. These results are consistent with previously published work in other common Acer tree species [8, 34] but it also includes species never tested before, some of which are known to contain substances that are toxic to equids and ruminants [42–45]

In conclusion, this work describes the successful validation of an improved and novel LC-MS method for detection of HGA in plant material that does not require the use of chemical derivatisation to produce accurate, reliable and reproducible results. The analytical method was successfully tested in sycamore tree material and using material from several other tree and plant species as negative controls. Additionally, the study showed that *A*. *pseudoplatanus* is likely the only source of HGA among other common trees encountered in European pastures.
